# Giant Cell Arteritis and Polymyalgia Rheumatica: 2016 Update

**DOI:** 10.5041/RMMJ.10262

**Published:** 2016-10-31

**Authors:** Gideon Nesher, Gabriel S. Breuer

**Affiliations:** Department of Internal Medicine A and the Rheumatology Unit, Shaare-Zedek Medical Center, Jerusalem, Israel

**Keywords:** Duplex ultrasonography, glucocorticoids, headache, temporal arteries, temporal artery biopsy, vision loss

## Abstract

Giant cell arteritis (GCA) and polymyalgia rheumatica (PMR) are both more common among people of North European decent than among Mediterranean people. Women are 2–3 times more commonly affected. Giant cell arteritis and PMR are extremely rare before age 50 years. Polymyalgia rheumatica may be “isolated” or associated with GCA. There is increased expression of inflammatory cytokines in temporal arteries of PMR patients, without overt histological evidence of arteritis. One-third of “isolated” PMR patients have vascular uptake in positron emission tomography (PET) scans, suggesting clinically unrecognized, “hidden” GCA. Typical manifestations of GCA are headache, tenderness over temporal arteries, jaw claudication, PMR, acute vision loss, and low-grade fever. Bilateral aching of the shoulders with morning stiffness is typical for PMR. In both conditions sedimentation rate and C-reactive protein are elevated, and anemia and thrombocytosis may occur. Color duplex ultrasonography of the temporal arteries may aid in GCA diagnosis. Temporal artery biopsy showing vasculitis, often with giant cells, confirms GCA diagnosis. In cases with negative biopsy one must rely on the clinical presentation and laboratory abnormalities. The diagnosis of PMR is made primarily on clinical grounds. Other conditions that may mimic GCA or PMR must be excluded. Glucocorticoids are the treatment of choice for both conditions. Prompt treatment is crucial in GCA, to prevent irreversible complications of acute vision loss and stroke. Addition of low-dose aspirin may further prevent these complications. The average duration of treatment is 2–3 years, but some patients require a prolonged course of treatment, and some may develop disease-related or treatment-related complications. No steroid-sparing agent has been proven to be widely effective thus far, but some promising therapeutic agents are currently being studied.

## INTRODUCTION

Giant cell arteritis (GCA) is a form of vasculitis that involves the major branches of the aorta with predilection for the vertebral, subclavian, and the extracranial branches of the carotid arteries, including the temporal arteries. The aorta and other large and medium-sized arteries may also be involved. Giant cell arteritis develops in individuals older than 50 years of age, and its incidence increases with age. It is considered to be the most common type of vasculitis in this age group.

## EPIDEMIOLOGY

Giant cell arteritis is more common among North Europeans, reaching an annual incidence rate above 20 cases per 100,000 population at risk (age >50). It is less common among Mediterranean people. In Israel, the incidence is similar to other Mediterranean countries.^1^–^2^ The incidence of GCA seems to have been decreasing in the last two decades.^1^–^4^ Women are affected 2–3 times more commonly than men.

Polymyalgia rheumatica (PMR) patients share many epidemiologic and pathogenetic features with GCA. Like GCA, PMR develops in patients older than 50 years and is more common in women. Similar to GCA, the highest annual incidence rates were observed in Northern Europe, 50–100 per 100,000 population older than 50 years. It is unknown whether PMR is just an expression of an underlying GCA. More likely, it seems that both are a result of an unknown causative factor (or factors), sometimes expressed as PMR, sometimes as GCA, and sometimes as a combination of both conditions. There is a wide range of the reported frequency of PMR in GCA patients (17%–66%), and it is estimated that 10% of “isolated” PMR cases develop GCA symptoms.^5^ Clinically, the two conditions may present together but may sometimes be separated by long intervals, and either one may present first.

## ETIOLOGY AND PATHOGENESIS

Etiology is unknown. Several studies implicated infectious agents such as varicella-zoster virus,^6^ but DNA sequencing of temporal artery biopsy specimens from GCA cases showed no evidence of previously suspected pathogens.^7^ The pathogenesis of GCA has been extensively studied but is still not fully understood. It is considered to be a T cell-dependent disease. Upon dendritic cell activation in the adventitia by an unknown antigen, CD4 T cells are recruited and polarized into Th1 and Th17 lines, producing interferon (IFN)-γ and interleukin (IL)-17, respectively, as their main cytokines. The production of cytokines and activation of macrophages and vascular smooth muscle cells induce systemic manifestations, vascular remodeling, and local ischemic manifestations.^8^–^10^ Macrophages, often forming giant cells, are the major source of cytokines, growth factors, and metalloproteinases.

There is evidence for increased expression of inflammatory cytokines in the temporal arteries of PMR patients, without overt histological evidence of arteritis. There is local expression of IL-1, IL-2, IL-6, and transforming growth factor (TGF)-beta.^11^ However, in contrast with GCA, T cells producing IFN-γ are not attracted to the vessel wall. This lack of IFN-γ expression in temporal arteries from PMR patients suggests that its production is crucial to the development of overt vasculitis and that a Th1 response may be suppressed in PMR. Using positron emission tomography (PET) scans, increased uptake was documented in thoracic blood vessels in one-third of apparently “isolated” PMR patients, suggestive of inflammation in these vessels,^12^,^13^ or clinically unrecognized, “hidden” GCA.

## CLINICAL FEATURES

The signs and symptoms of GCA may be classified into four subsets: signs and symptoms of cranial arteritis, extracranial arteritis, systemic symptoms, and PMR. Patients may develop any combination of these manifestations. In most instances symptoms develop gradually over a period of several weeks, but onset may sometimes be abrupt.

Headache is a frequent presenting symptom and is typically felt over one or both temporal areas. The temporal arteries may seem prominent and tender. Generalized or occipital headaches are not uncommon. The headache may be continuous or paroxysmal. Pain in the jaw during mastication, facial pain, and scalp tenderness may also be present. Rarely, GCA may lead to segmental scalp necrosis or tongue infarction.

Neurologic manifestations are uncommon. Strokes occur in 3%–7% of GCA patients.^14^ Involvement of the vertebro-basilar system is relatively more common in GCA-related strokes than in atherosclerotic strokes.^14^,^15^ It is important to note that strokes may still develop after glucocorticoid therapy is begun.^14^,^16^,^17^ Neuropsychiatric manifestations and peripheral neuropathies are uncommon.

Arteritic anterior ischemic optic neuropathy (A-AION), developing in 5%–15% of the patients, is the leading cause of blindness in GCA, sometimes being the presenting manifestation.^18^,^19^ Arteritic AION is unilateral in most cases, but visual loss in the other eye may develop; A-AION results from vasculitis of the posterior ciliary arteries, branches of the ophthalmic artery, which supply the optic nerve head and the choroid. Less common causes of visual loss are central retinal artery occlusion, posterior ischemic optic neuropathy, and cortical blindness. Vision loss in GCA is sudden. However, several studies reported that 50% or more of GCA patients with irreversible visual loss had premonitory visual symptoms, such as blurry vision, amaurosis fugax, visual hallucinations, or diplopia.^14^,^18^ Amaurosis fugax is the most ominous sign of impending visual loss. It should be considered a medical emergency in GCA, as prompt treatment with glucocorticoids and low-dose aspirin may prevent the development of irreversible blindness. Jaw claudication is also associated with increased risk of developing vision loss.^20^ Following vision loss, the visual outcome is poor, even in patients treated with high doses of steroids. Only 5% had some improvement of both visual acuity and central visual field.^21^

Vestibulo-auditory manifestations are quite common in prospective studies.^22^ Symptoms are unilateral or bilateral hearing loss, vertigo, and tinnitus. Onset is usually insidious. A total of 89% of GCA patients had abnormal vestibular tests, 64% had subjective hearing impairment, 52% had vertigo, and 50% had tinnitus. These were reversible in most cases following steroid therapy.

Signs of occlusive changes in large arteries of the chest and extremities are uncommon. The clinical findings are those of the aortic arch syndrome, indistinguishable from those of Takayasu arteritis: arm claudication, Raynaud’s phenomenon, absent or decreased pulses, and bruits over the involved arteries. Clinical signs of aortitis are uncommon.^23^ However, this condition is under-diagnosed, as symptoms are frequently mild, non-specific, and insidious. It is estimated that GCA patients have a 2-fold increased risk of aortic aneurysm.^24^ Symptoms suggestive of involvement of the coronary, mesenteric, and lower extremity arteries are rare.

Systemic manifestations (fever, malaise, fatigue, anorexia, and weight loss) occur quite often, in 30%–60% of the patients. In some cases, these may be the only symptoms of GCA. Fever is typically low-grade, and temperatures rarely exceed 39°C. The typical symptoms of PMR are aching of the shoulder girdle and neck, associated with morning stiffness. The hip girdle may also be involved. These symptoms are probably related to inflammation of the glenohumeral and hip joints, and the subacromial, subdeltoid, and trochanteric bursae. One-third of PMR patients have systemic manifestations such as low-grade fever, malaise, and anorexia, but these are milder than in GCA. A subgroup of PMR patients presents with arthralgia or synovitis of peripheral joints, predominantly hands and knees.^25^ Some present with symmetric synovitis with pitting edema, compatible with the relapsing seronegative symmetric synovitis with pitting edema (RS_3_PE) syndrome.^26^ It is difficult at times to distinguish between these patients and patients with elderly-onset rheumatoid arthritis (RA).^27^

## DIAGNOSIS

The diagnosis of PMR and GCA is made primarily on clinical grounds and is bolstered by laboratory evidence of an acute-phase reaction, most commonly elevated levels of erythrocyte sedimentation rate (ESR) and C-reactive protein (CRP), as well as anemia of inflammation and thrombocytosis. Various conditions can mimic GCA and PMR,^28^ and should be considered in the differential diagnosis (Table 1). A small group of patients may present with typical features of the disease but without elevation of the ESR.^29^

**Table 1:** Conditions That Should Be Considered in the Differential Diagnosis of PMR and GCA.

Polymyalgia Rheumatica (PMA)	Giant Cell Arteritis (GCA)
Elderly-onset rheumatoid arthritis	Sinusitis
Fibromyalgia	Dental and temporo-mandibular conditions
Shoulder bursitis/tendinitis	Non-arteritic anterior ischemic optic neuropathy
Cervical spondylosis	Subacute thyroiditis
Ankylosing spondylitis/sacroileitis (early stages)	Chronic infections (infective endocarditis, etc.)
Hypothyroidism	Trigeminal neuralgia
Viral infections, chronic infections	Malignancy
Polymyositis	Atherosclerotic cardiovascular disease
Malignancy	
Amyloidosis	

**Figure 1:**
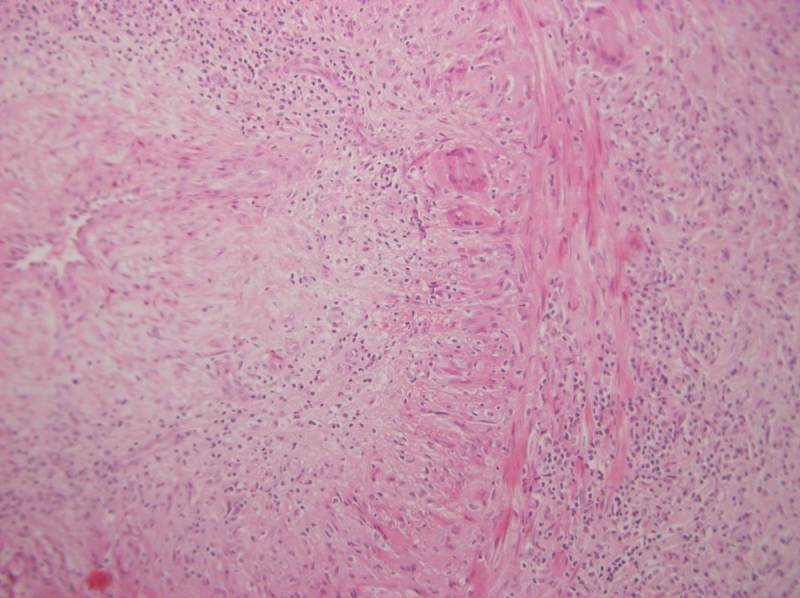
Temporal Artery of a Patient with Giant Cell Arteritis. Image shows intense trans-mural inflammatory infiltrate, multi-nucleated giant cells, intimal hyperplasia, and severe narrowing of the lumen.

The only test that confirms the diagnosis of GCA is a temporal artery biopsy, showing vasculitis with mononuclear cell infiltrates, often with giant cells (Figure 1). Giant cell arteritis affects the vessels in segments, therefore areas of vasculitis may be missed and the histological examination is normal in 10%–30% of GCA patients (“biopsy-negative GCA”).^30^ A temporal artery biopsy length larger than 5 mm is associated with increasing diagnostic yield, but the optimal length is probably higher.^31^ Biopsying temporal arteries on both sides may also increase the diagnostic yield.^32^ It is preferable to perform the biopsy as soon as possible, but the specimen may show signs of arteritis even after 2–4 weeks of treatment.^33^

**Figure 2:**
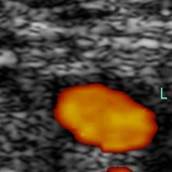

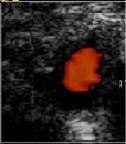
Ultrasonography of Temporal Artery of a Patient with Giant Cell Arteritis. **Left:** Normal duplex ultrasonography of the left temporal artery of a patient with giant cell arteritis. **Right**: Duplex ultrasonography of the right temporal artery of the same patient, showing peri-luminal dark halo.

Some imaging modalities may aid in the diagnosis of GCA. Among those, color duplex ultrasonography of the temporal arteries is more commonly used. A peri-luminal hypo-echoic halo (Figure 2), probably representing vessel-wall edema, was suggested to be highly sensitive and specific for GCA.^34^ However, results are operator-dependent and vary considerably among studies examining the diagnostic value of color duplex ultrasonography.^35^

High-resolution contrast-enhanced magnetic resonance imaging (MRI) of the temporal arteries also enables evaluation of possible inflammation of the vessel wall. Preliminary results show high sensitivity of this imaging modality,^36^ with diagnostic yield comparable to color duplex ultrasonography.^37^ Angiography of the aortic arch and its branches may serve to diagnose large-vessel involvement. Non-invasive modalities, such as PET scans, may also be employed to detect large-vessel involvement in the chest, neck, and abdomen.^38^ The specificity of PET for GCA diagnosis is considered to be very high (~100%), but sensitivity is lower (~66%). It may also serve to evaluate response to treatment and GCA disease exacerbations.

Serological tests were not helpful in diagnosing GCA. Autoantibodies were not consistently found in GCA, although plasma cells can be found in the adventitia in 7%–24% of temporal artery biopsies from patients with GCA.^39^ The exception was antiphospholipid antibodies (APLA), which were found in 30%–80% of GCA cases.^40^–^43^ Antibodies to lamin C were found in one-third of GCA patients and in none of the controls.^44^ Autoantibodies to a human ferritin peptide (the heavy chain N-terminal) were found in 92% of 36 patients with GCA and/or PMR.^45^,^46^ In addition, 89% had antibodies to bacterial ferritin peptide of *Staphylococcus epidermidis*. Anti-ferritin antibodies were found in much lower rates in disease controls. Following these reports, another group of researchers reported their experience with anti-ferritin antibodies in GCA.^47^ They found a test sensitivity of 82% in biopsy-positive GCA patients. Anti-ferritin antibodies were found in 34% of disease controls and 3% of healthy controls. Upon further testing, these antibodies may prove useful as a diagnostic marker of GCA.

There are no independent validating criteria to determine whether GCA is present when a temporal artery biopsy is negative. The American College of Rheumatology (ACR) criteria for classification of GCA^48^ may assist in GCA diagnosis (Table 2). However, those classification criteria serve mainly to classify GCA within the group of vasculitides. Their purpose was to differentiate GCA from other types of vasculitis, and not to differentiate GCA from other disease conditions. It is highly important to realize that these are not diagnostic criteria. Their validity as criteria for GCA diagnosis has been questioned.^49^ Such classification criteria do not work well when used for diagnosing individual cases.^50^ The final diagnosis should be based on all clinical, laboratory, imaging, and histological findings.

**Table 2:** The American College of Rheumatology 1990 GCA Classification Criteria.

GCA Classification Criteria
(1) Age at onset ≥50 years
(2) A new headache
(3) Temporal artery abnormality such as tenderness to palpation or decreased pulsation
(4) Erythrocyte sedimentation rate ≥50 mm/h
(5) Abnormal artery biopsy showing vasculitis with mononuclear cell or granulomatous inflammation, usually with giant cells

At least three of the five parameters must be present, which yields a sensitivity of 93% and a specificity of 91%, in relation to controls with other vasculitides.

The diagnosis of PMR is made primarily on clinical grounds and laboratory evidence of an acute phase reaction.^28^,^51^ There is no single diagnostic test for PMR, but sets of diagnostic and classification criteria have been suggested by several groups of investigators. Recently, provisional classification criteria (Table 3) were published as a collaborative initiative of the European League Against Rheumatism (EULAR) and the ACR.^52^ With these criteria, the required score has 68% sensitivity and 78% specificity for discriminating PMR from comparison patients. The positive predictive value is 69%, and the negative predictive value 77%. These new classification criteria need further validation.

**Table 3:** The 2012 Provisional PMR Classification Criteria (A Collaborative Initiative of the European League Against Rheumatism and the American College of Rheumatology).

PMR Classification criteria	Points
**Clinical Criteria**	
Morning stiffness duration >45 min	2
Hip pain or limited range of motion	1
Absence of rheumatoid factor or anti-citrullinated peptide antibodies	2
Absence of other joint pain	1
**Ultrasound Criteria**	
At least one shoulder with subdeltoid bursitis and/or biceps tenosynovitis and/or glenohumeral synovitis (either posterior or axillary), and at least one hip with synovitis and/or trochanteric bursitis	1
Both shoulders with subdeltoid bursitis, biceps tenosynovitis, or glenohumeral synovitis	1

Required criteria for all cases: (1) age 50 or older; (2) bilateral shoulder pain; (3) elevated ESR and/or CRP.

With only clinical criteria, a score of >4 is required. With combined clinical and ultrasound criteria, a score of >5 is required. The required score has 68% sensitivity and 78% specificity for discriminating PMR from comparison patients. The positive predictive value is 69%, and the negative predictive value 77%.

There is a wide range of the reported frequency of GCA in patients presenting with PMR. One diagnostic option is routinely to biopsy the temporal arteries in all PMR patients. In this approach, the chance of missing GCA is small, but the frequency of positive biopsies is very low.^53^ The more accepted strategy is to biopsy only those patients who have symptoms suggestive of GCA. With this approach the results are likely to vary according to the expertise of the examining physician.^54^ There have been some attempts to develop guidelines for performing temporal artery biopsy in patients with PMR.^55^,^56^ Patients with PMR who were younger than 70 years of age, and with no features of cranial vasculitis (such as a new headache, jaw claudication, abnormalities of the temporal arteries on examination, or amaurosis fugax), were unlikely to have a positive biopsy. Older patients with cranial vasculitis findings were more likely to have a positive biopsy. Severe degrees of anemia and thrombocytosis are suggestive of GCA in patients presenting with PMR symptoms. Also, poor clinical response to low-dose prednisone (15–20 mg/day) and persistent abnormalities in laboratory parameters of inflammation are also suggestive of GCA in patients presenting with “isolated” PMR. In such cases ultrasonography and biopsy of the temporal arteries should be performed to rule out GCA.

## THERAPY, DISEASE COURSE, AND PROGNOSIS

Glucocorticoids are the mainstay of treatment for both PMR and GCA.^57^,^58^ In PMR, the starting recommended dose is 15–20 mg/day. Symptoms typically begin to abate within 1–3 days, but a few patients may still have some pain and stiffness several weeks after initiation of treatment. After 2–4 weeks, following improvement of the clinical features of the disease together with normalization of the inflammatory markers, the dose of glucocorticoids can be tapered gradually. Relapses occur in about one-half of the patients, with response to increasing the dose for several weeks. Tapering of the dose should then be resumed.

The duration of treatment for PMR varies from 6 months to several years, and the majority of patients develop steroid-related side effects. Methotrexate was evaluated as a steroid-sparing agent with mixed results. Addition of methotrexate can be considered in relapsing disease, in patients who are regarded at high risk for developing adverse events, or in patients experiencing steroid-related adverse effects.^59^ Polymyalgia rheumatica is thought to have a benign course, with a variable degree of treatment-related morbidity.

In GCA, the initial dose is 40–60 mg/day for most cases.^58^ Patients with vascular complications such as vision loss (transient or permanent), diplopia, transient ischemic attacks, or stroke may be treated initially with higher doses such as 500–1,000 mg/day of intravenous methylprednisolone for three consecutive days, in an attempt to prevent additional ischemic complications.

Rapid improvement of all clinical manifestations following treatment initiation is characteristic. Prompt treatment is crucial in GCA to prevent irreversible ischemic complications, such as acute vision loss or stroke. Thus therapy should not be delayed pending temporal artery biopsy, which should be performed as soon as possible.

After 2–4 weeks, following improvement of the clinical features of the disease together with normalization of ESR and CRP, the dose of glucocorticoids can be tapered, with close monitoring for recurrence of symptoms. During follow-up, levels of ESR and CRP do not always correlate with disease activity. Elevation of these acute-phase reactants while the patient is asymptomatic is not an absolute indication to increase the dose of prednisone. In such cases, it is preferable to slow the rate of dosage tapering and continue to watch closely for recurrence of symptoms. On the other hand, the dose should be increased if symptoms recur, even when the ESR or CRP remain within the normal range. The average duration of treatment is 2–3 years. Relapses are experienced by about half of GCA patients. Most relapses are mild, but some patients may still develop vision loss or stroke during the course of corticosteroid treatment or after discontinuation of therapy. Addition of low-dose aspirin (100 mg/d) has been shown to decrease the rate of vision loss and stroke during the course of the disease, probably mediated by its anti-platelet effect.^60^,^61^

Some GCA patients with thoracic or peripheral vascular involvement may benefit from surgical interventions, such as balloon angioplasty or stents for vascular stenosis, and prostheses for aortic aneurysms. Aortic complications may occur early or late in the course of GCA, sometimes after the completion of drug treatment. In most cases, aortic structural damage was not associated with persistence of detectable disease activity.^62^ Indications for regular imaging of the aorta in GCA have not yet been determined.^63^

Individual cases vary greatly, therefore the exact doses and the duration of treatment should be adjusted to the needs of the individual patient, considering both disease manifestations and glucocorticoid adverse effects. Patients with strong initial systemic inflammatory response tend to have a prolonged disease course with more flares, requiring higher cumulative steroid doses.^64^ No steroid-sparing agent has been proven to be highly effective thus far, but a few reports suggest some beneficial effects of methotrexate and cyclophosphamide.^65^,^66^ These medications may be prescribed in certain situations, such as resistant cases, or in cases at high risk of glucocorticoid-related side effects. Recently, several case studies reported beneficial effects of tocilizumab, an IL-6 receptor antagonist. A randomized prospective study of 30 patients reported recently that patients given a combination of tocilizumab and prednisolone were able to discontinue prednisolone 12 weeks earlier than patients given prednisolone only.^67^

It is apparent that GCA may result in fatal complications. The major causes of mortality have been vascular: stroke, coronary artery events, rupture of thoracic aortic aneurysms, and aortic dissection. In addition, there is increased occurrence of severe infections.^68^ The impact on the overall prognosis is controversial.^2^,^3^,^68^–^70^ Some epidemiologic studies reported increased mortality, mostly during the first year after diagnosis, while others found that the overall life expectancy among GCA patients was essentially identical to that of the general population.

## CONCLUSIONS

Giant cell arteritis is considered to be the most common type of primary vaculitis in the elderly. It may cause severe morbidity such as acute loss of vision and stroke, but prompt diagnosis and proper treatment would prevent such ischemic episodes in most cases. It is unknown whether PMR is just an expression of an underlying GCA. More likely, it seems that both are a result of an unknown causative factor (or factors), sometimes expressed as PMR, sometimes as GCA, and sometimes as a combination. Clinically, the two conditions may present together but may sometimes be separated by long intervals, and either one may present first. Treatment-related morbidity is a concern in both conditions, but several promising therapeutic agents are currently being studied.

## Abbreviations

CRP: C-reactive protein
ESR: elevated sedimentation rate
GCA: giant cell arteritis
IL: interleukin
INF: interferon
PET: positron emission tomography
PMR: polymyalgia rheumatica
RS_3_PE: relapsing seronegative symmetric synovitis with pitting edema
